# How can human values influence work engagement among teachers? an exploratory study

**DOI:** 10.1007/s43076-023-00258-y

**Published:** 2023-02-08

**Authors:** Gabriel Lins de Holanda Coelho, Patrícia Nunes da Fonsêca, Roosevelt Vilar, Luis Augusto de Carvalho Mendes, Valdiney Veloso Gouveia

**Affiliations:** 1 University College Cork, Cork, Ireland; 2 Federal University of Paraíba, Joao Pessoa, Brazil; 3 Universitas Sebelas Maret, Surakarta, Indonesia; 4 Faculdade Maurício de Nassau, João Pessoa, Brazil

**Keywords:** Work engagement, Human values, Teachers, Motivation

## Abstract

Human values provide essential insights into how personal characteristics can help build a more positive work environment within an educational context, impacting variables such as organizational commitment and satisfaction with university life. However, it is still unknown to what extent values can help to influence the teachers’ level of work engagement. That is, whether specific values can lead people to present a higher motivation at work. Therefore, we conducted one study (*N* = 345; Mage = 36.45, SDage = 10.33) to assess teachers’ associations between human values and work engagement. We used the Basic Values Survey and the Utrecht Work Engagement Scale. To evaluate the data, we checked the associations between the variables using Pearson’s correlations and whether human values could significantly predict work engagement using hierarchical regressions. Our results showed that all values significantly correlate to the work engagement dimensions. However, only interactive values (e.g., affectivity, belonging, support), characterized by the motivation to develop and maintain relationships with others, significantly predicted work engagement. Such findings highlight the importance of an environment that allows the development and maintenance of relationships between teachers and their peers and students, favoring a more excellent state of mind towards their work and enhancing their motivation to do their job. We are confident that our research brings novelty to the literature on work engagement, providing the first assessment of human values’ impact on teachers’ motivation towards work.

## Introduction

Values play an important role in understanding human behavior (Maio, 2016). Researchers have significantly evaluated their associations with a range of variables, such as age and gender (Vilar et al., 2020), well-being (Hanel et al., 2020), and preference for economy or health during the COVID-19 pandemic (Coelho et al., 2021). More specifically, their role has also been studied within an educational setting. For instance, benevolent (e.g., responsible, helpful) and conformity (e.g., self-discipline, politeness) values significantly and positively impact teachers’ organizational commitment (Cohen, 2010). These values also positively influence satisfaction with university life in students’ (Ng & Ye, 2016) and teachers’ self-efficacy (Barni et al., 2019). Therefore, human values clearly contribute to understanding the intrinsical psychosocial mechanisms within an educational context, from both teachers’ and students’ perspectives. Values provide insights into how personal characteristics can help build a more positive work environment. However, studies examining the extent to that values can predict work engagement are still scarce (Dyląg et al., 2013). Therefore, in this exploratory study, we aim to understand better how human values can act as motivators of work engagements among teachers in Brazil.

### Work engagement

The study of work engagement only gained greater attention recently, with the prominence of positive psychology (Schaufeli, 2013). This new focus allowed a more in-depth understanding of the workplace processes and how these can influence the employees’ journey. Work engagement is commonly measured through three dimensions (Schaufeli et al., 2002): (1) vigor, characterized by high levels of energy and resilience while working, with employees willing to invest time in their work, even through challenging moments; (2) dedication, characterized by a sense of significance, enthusiasm, inspiration, pride and challenge; and (3) absorption, characterized by a deep concentration and involvement at work, where the employee has difficulties to let go of it.

Through the years, researchers have been interested in how work engagement functions within an educational setting. For instance, through multi-level analyses, Bakker and Bal (2010) found that work engagement is positively associated with variables such as level of autonomy, opportunities for development, and job resources and that these impact job performance. Similarly, work engagement significantly mediated the relations between job resources and organizational commitment (Hakanen et al., 2006), i.e., more resources result in greater work engagement and higher organizational commitment. Finally, teachers with greater self-efficacy also presented higher engagement levels and experienced more feelings such as joy, pride, and love (Burić & Macuka, 2018). Despite these significant findings, it is vital to highlight the importance of a more in-depth understanding of how individual psychological variables can impact work engagement. More specifically, it is essential to contribute to the scarce literature examining whether specific motivational goals, such as human values, can impact how teachers engage in their work. For instance, can specific human values be more intrinsically connected to the different types of work engagement?

### Values and work engagement

Values can be defined as abstract ideas that represent our motivational goals (Schwartz, 2012). For instance, some individuals might be driven by values such as promotion and equality, and whether their work environment facilitates the satisfaction of these values might help to explain their motivation to work. An environment that allows employees to thrive and progress in their careers would help to satisfy values such as promotion. On the other hand, an environment that promotes fairness among the employees would help to commit those endorsing equality values.

In our study, we used the Functional Theory of Human Values (Gouveia et al., 2014), which presents values into a 2 × 3 framework based on their underlying needs (i.e., materialistic or humanitarian) and goals (i.e., personal, central, or social). This framework results in six value subfunctions (Gouveia et al., 2008, 2014): (1) excitement (examples of values: *emotion*, *pleasure*), which refers to the needs for variety and pleasurable situations; (2) suprapersonal (e.g., *knowledge*, *maturity*), concerning the need for aesthetics, cognition, and self-actualization; (3) interactive (e.g., *support*, *belonging*), characterized by values that represent the development and maintenance of relationships; (4) promotion (e.g., *power*, *success*), representing motivations for personal and material achievements; (5) existence (e.g., *stability*, *survival*), covering the need for primary conditions for individual’s survival; and (6) normative (e.g., *obedience*, *tradition*), representing the seek for security and control. The 2 × 3 framework can be seen in Table 1.

**Table 1:** The functional theory of human values 2 × 2 framework

		Values as guides of actions (circle of goals)		
		Personal goals	Central goals	Social goals
Values as expressions of needs (level of needs)	*Thriving* *needs*	Excitement Values	Suprapersonal Values	Interactive Values
		Emotion	Beauty	Affection
		Pleasure	Knowledge	Belonging
		Sexuality	Maturity	Support
	*Survival* *needs*	Promotion Values	Existence Values	Normative Values
		Power	Health	Obedience
		Prestige	Stability	Religiosity
		Success	Survival	Tradition

Adapted from Gouveia et al. (2014)

The essential role in predicting human behavior (Maio, 2016) has brought a spotlight to the study of values within the organizational context. For instance, studies have shown that when individuals perceive that they value similar things as the institution they work in, they experience more robust work engagement (Li et al., 2015). Meta-analyses have also supported the positive impact of this person-organization fit on work attitudes (e.g., Kristof-Brown et al., 2005; Verquer et al., 2003). Finally, another important study on values and work engagement was conducted by Dylag et al. (2013), which assessed how value discrepancy contributes to the explanation of work engagement and job burnout. The authors found that values with collective interests are more strongly connected to vigor, dedication, and absorption than values with personal and mixed interests.

Despite the significant findings about the relationship between values and work engagement, these studies mentioned above are not without limitations. For instance, Li et al. (2015) measure values in a superficial (e.g., “The things that I value in life are very similar to the things that my school values”). Such measurement does not account for the diversity of values that modern value theories have conceptualized (Gouveia et al., 2014; Schwartz, 1992). Another limitation can be seen in Dylag et al. (2013) study, in which they use higher-order values that collapse specific value factors (Schwartz & Bilsky, 1987), which might hide significant findings (Vilar et al., 2020). Therefore, a closer look at these relations is needed.

### The present research

In this exploratory research, we used a correlational design to assess how human values relate to and impact work engagement among teachers from Brazil. We used Pearson’s correlations and hierarchical regressions, with values as predictors of the three engagement dimensions (vigor, dedication, and absorption). We also controlled these regressions for the effect of variables commonly known to affect work engagement, such as work capacity, work conditions, and work satisfaction. Despite the exploratory nature, some significant associations can be expected based on the motivational nature of the six subfunctions of the Functional Theory (see Table 1). For instance, promotion values, characterized by pursuing personal and material achievements (Gouveia et al., 2014), could be an important motivator to help teachers’ higher work engagement. If a teacher has long-term goals that require either monetary gains or to “climb the ladder” within the company, promotion values might help keep them more involved with the work. Another example is the interactive values concerning the development of social relationships (Gouveia et al., 2014), which could also significantly impact how teachers perceive their workplace. A more social environment with solid relationships could help satisfy teachers’ motivations and make them feel more welcome and engaged.

## Method

### Participants and procedure

Participants were voluntarily invited to answer the survey through social media (e.g., Facebook, Instagram). The researchers advertised the survey link on these online platforms, informing participants of the study goals, its voluntary character, and ethical considerations. Emails were made available in case participants had any queries.

Participants were 345 teachers from Brazil, with a mean age of 36.45 (SD = 10.33), and mainly women (*n* = 252; 73.9%). These teachers had an average experience of 11.3 years (SD = 8.74), worked 31.2 h weekly (SD = 12.5) in a public institution (56.2%), and had an average salary of 3.200,83 *Brazilian Reais* (SD = 2.602,75). They give classes to either a specific educational level (79.7%), such as high school (14.6%) and undergraduates (10.4%), or multiple educational levels (e.g., both elementary and high school, 11.9%). No missings were reported.

### Material

*Basic Values Survey* (Gouveia et al., 2008) is a measure composed of eighteen items equally distributed among six subfunctions: excitement (e.g., *emotion*), suprapersonal (e.g., *knowledge*), interactive (e.g., *support*), promotion (e.g., *power*), existence (e.g., *stability*), and normative (e.g., *obedience*). Participants indicate to what extent each item is important to them, using a seven-point scale (1 = *completely unimportant*; 7 = *of the utmost importance)*. The internal consistencies in this study were in line with prior research (Gouveia et al., 2014; Schwartz, 1992; Vilar et al., 2020), ranging from a McDonald’s omega of 0.45 (excitement) to 0.66 (existence), and a Cronbach’s alpha of 0.44 (excitement) to 0.66 (existence). The whole questionnaire presented an omega of 0.82 and an alpha of 0.83. All reliability levels are available in Table 2.

**Table 2:** Correlations between work-related variables, work engagement, and human values

		*M*	*SD*	α	ω	1	2	3	4	5	6	7	8	9	10	11
1	Work capacity	8.45	1.12													
2	Work conditions	7.24	1.94			.357**										
3	Work satisfaction	7.79	1.92			.408**	.594**									
	*Work engagement*			.90	.90											
4	Vigor	4.56	1.01	.79	.80	.325**	.281**	.481**								
5	Dedication	5.10	.88	.77	.78	.353**	.325**	.511**	.753**							
6	Absorption	4.57	.94	.71	.71	.244**	.252**	.420**	.742**	.720**						
	*Human values*			.83	.82											
7	Excitement	5.20	.95	.44	.45	.037	.074	.063	.078	.119*	.078					
8	Suprapersonal	6.01	.73	.52	.53	.038	-.057	.028	.096	.146**	.127*	.316**				
9	Interactive	5.98	.84	.61	.62	.021	.019	.055	.195**	.202**	.190**	.312**	.601**			
10	Promotion	5.05	.91	.45	.46	.079	.039	.087	.149**	.152**	.107*	.427**	.339**	.305**		
11	Existence	6.27	.80	.66	.66	.062	.010	.017	.109*	.153**	.152**	.378**	.599**	.582**	.367**	
12	Normative	5.90	.87	.52	.53	.146**	.203**	.113*	.164**	.177**	.141**	.205**	.382**	.404**	.244**	.521**

*α* Cronbach’s alpha, *ω* McDonald’s omega; *p < .05, **p < .01

*Utrecht Work Engagement Scale* (Schaufeli et al., 2002) is a measure composed of 17 items that are distributed among three dimensions: vigor (e.g., *At my job, I am very resilient, mentally*), dedication (e.g., *I am proud on the work that I do*), absorption (e.g., *time flies when I’m working*). Participants indicate how often they experience these different situations within their workplace, using a seven-point scale (0 = *never*; 6 = *every day*). Reliability levels ranged from 0.71 (absorption) to 0.80 (vigor) for mcdonald’s omega, and from 0.71 (absorption) to 0.79 (vigor) for Cronbach’s alpha. The whole questionnaire presented omega and alpha of 0.90. All reliability levels are available in Table 2.

Moreover, to assess the unique impact of human values on work engagement, we controlled the analyses for work-related variables (i.e., work capacity, work conditions, work satisfaction) known for being associated with work engagement. Participants answered three single items to assess these different aspects, using a scale from 0 to 10.

### Data analysis

The relatively large sample in our study allows the performance of these parametric analyses without the need to perform normality tests (see Field, 2013; Ghasemi & Zahediasl, 2012). We also expect a linear relationship between them — e.g., the increase of one variable is linked to the increase of the other. All analyses were performed using SPSS. We performed Pearson’s correlations between the value subfunctions, work engagement, and the additional work-related variables. Further, we performed hierarchical regressions, with the work-related variables and values predicting the three dimensions of work engagement.

## Results

First, we performed Pearson’s correlations between the variables. As shown in Table 2, the three work engagement dimensions were significantly and positively correlated to all work-related variables and most human values. The exceptions were excitement, which was only correlated with the dedication dimension, and suprapersonal, correlated only with dedication and absorption. However, as all values were significantly related to at least one work engagement dimension, we decided to include them in the next analyses.

**Fig. 1:**
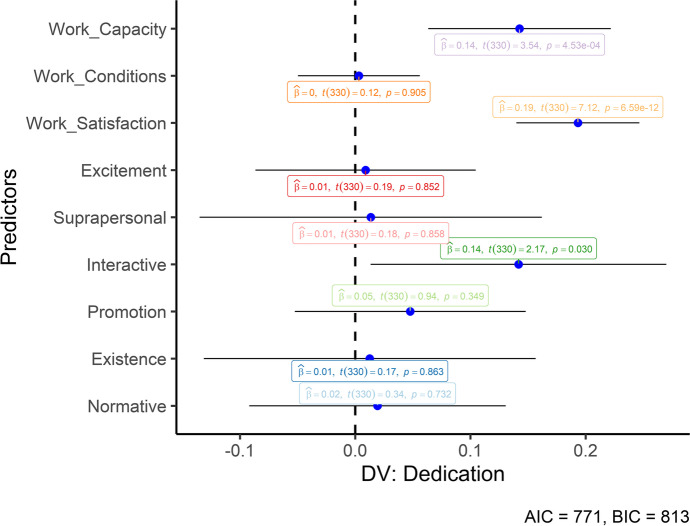
Unstandardized regression coefficients (Vigor). Error bars represent 95% CIs

**Fig. 2:**
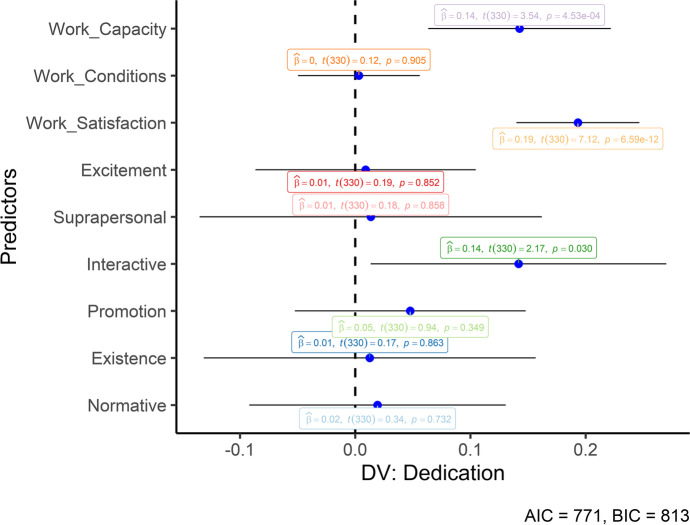
Unstandardized regression coefficients (Dedication). Error bars represent 95% CIs

**Fig. 3:**
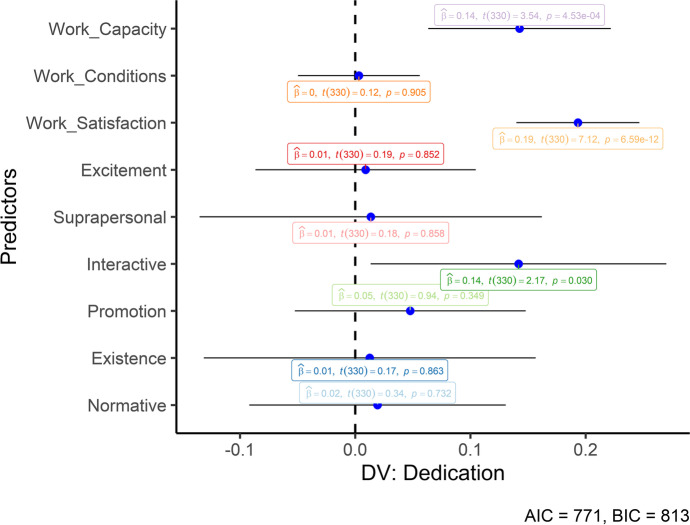
Unstandardized regression coefficients (Absorption). Error bars represent 95% CIs

After, we performed multiple Hierarchical Regressions. We added the work-related variables in the first step of the regressions to control their potential effect. In the second step, all six value subfunctions were included. The three work engagement dimensions were then included as the dependent variables. No multicollinearity was observed between our predictors (VIF < 10). The results of the hierarchical regressions can be seen in Table 3. Work satisfaction significantly predicted all three work engagement dimensions, whereas work capacity predicted vigor and dedication. Only interactive values significantly predicted the three dimensions of work engagement. However, this impact was significant beyond the controlled variables only for the vigor and dedication dimensions (i.e., significant *ΔR*^*2*^). Figures 1, 2 and 3 also graphically depicts the regressions (unstandardized) to help understand the predictors’ differences.

**Table 3:** Standardized regressions

		Work-related variables			Human values								
	M	Capacity	Conditions	Satisfaction	Exc	Sup	Int	Pro	Exi	Nor	*F(df)*	*R*^*2*^	*ΔR*^*2*^
Vigor	1	.160**	− .036	.437**							37.69 (3, 336)**	.25	
	2	.156**	− .045	.425**	− .017	− .055	.187**	.074	− .034	.046	14.80 (9, 330)**	.29	.04*
Dedication	1	.186**	.006	.432**							45.73 (3, 336)**	.29	
	2	.179**	.007	.419**	.010	.011	.134*	.049	.011	.019	17.55 (9, 330)**	.32	.03*
Absorption	1	.100	− .008	.384**							25.33 (3, 336)**	.18	
	2	.094	− .003	.375**	− .018	− .006	.134*	.021	.060	− .004	9.97 (9, 330)**	.21	.03

*M* model; *p < .05, **p < .01; *R*^*2*^ = amount of explained variance by the model; *ΔR*^2^ = increase in the *R*^2^ between the models

## Discussion

The study of work engagement in an educational setting has been extensively assessed over the years (e.g., Bakker & Bal, 2010; Burić & Macuka, 2018; Hakanen et al., 2006). However, studies providing a more in-depth picture of how psychological variables such as human values can impact work motivation are needed. Therefore, in the present research, we assessed how human values could influence work engagement. More specifically, we evaluated this impact considering a sample of teachers from Brazil, a context that represents challenges regarding infrastructure and low salaries (Sampaio et al., 2002), and a cultural environment focused on interpersonal relations (Hofstede, 2001).

All human values were significantly and positively correlated to the work engagement dimensions. More specifically, human values representing personal or social goals are strongly associated with vigor, dedication, and absorption. Personal values are typical in individuals focusing on the self (Gouveia et al., 2008). One example of personal value is promotion (e.g., *power*, *prestige*, *success*), commonly endorsed by individuals with a more substantial need for personal and material accomplishments (Gouveia et al., 2014). Previous research shows that promotion values have been significantly associated with work-related variables or economic aspects. For instance, during the COVID-19 pandemic, individuals that highly endorsed this value subfunction tended to prioritize the economy over the health of their fellow citizens (Coelho et al., 2021). That is, their intrinsic motivations towards achievements led them to present a higher motivation to work than others, despite the critical situation lived worldwide. However, we should consider that this preference for the economy (and consequently, work) during a pandemic does not necessarily reflect work engagement — many individuals might do it because they need the income to guarantee their basic needs. In another study, researchers found that such values significantly predicted gross margin return on investment and that high-involvement work practices mediated this relation (O’Neill et al., 2011). In other words, individuals motivated by personal and material accomplishments are more likely to get involved with the work practices of their organizations, as this involvement will help satisfy their needs. This higher involvement might help us understand the positive associations between personal values (e.g., promotion) and work engagement. When referring to teachers, those who endorse promotion values are more likely to present a higher engagement in their workplace, allowing them to fulfill their personal goals.

Moreover, values with a social goal are typical in individuals who focus on interpersonal relationships (Gouveia et al., 2014). In our study, both normative and interactive subfunctions were significantly associated with the work engagement dimensions. First, normative values (e.g., *obedience*, *tradition*) represent the seek for security and control (Gouveia et al., 2008). These positive associations with work engagement align with previous research, which found that values that share these characteristics significantly impact other work-related variables, such as organizational commitment (Cohen, 2010) and self-efficacy (Barni et al., 2019). Moreover, interactive values (e.g., *affectivity*, *belonging*, *support*) are characterized by the motivation to develop and maintain relationships with others (Gouveia et al., 2008). Importantly, this subfunction was the only one to significantly predict the three work engagement dimensions, highlighting the importance of endorsing such values to stimulate teachers within the workplace. These findings also align with prior research on workplace relationships’ benefits. For instance, in an educational setting, the relationships between teachers and students affect teachers’ professional and personal self-esteem (Spilt et al., 2011). In another study, employees’ satisfaction with their co-workers helped strengthen the influence of work-life balance on psychological well-being and job performance (Haider et al., 2018). Finally, this satisfaction with own co-workers was also related significantly to work engagement (Avery et al., 2007).

Additionally, it is important to highlight that the influence of interactive values on work engagement was controlled by work satisfaction and work capacity, which significantly predicted the dimensions of work engagement. When performing the hierarchical regressions, we assessed the unique impact of interactive values, controlling the variance that could account for work satisfaction and capacity.

## Limitations and future studies

Despite our significant findings, our study is not without limitations. First, due to the exploratory character of this study, we focused only on one psychological aspect that could influence work engagement, i.e., human values. Future studies could benefit from assessing the influence of a myriad of psychological mechanisms that, at an individual level, could impact engagement in the workplace, such as personality traits and needs. Second, we used a cross-sectional design, and therefore assessed the data at a single time point. A longitudinal assessment of the relationships between values and work engagement would be preferable. Third, we used self-report questionnaires, which might be subject to the influence of social desirability or response bias. Future studies could control for this potential impact. Finally, assessing participants’ daily demands and resources is essential to understand their work context better. Sampaio et al. (2002) have signalled that school infrastructure in Brazil is poor, and teachers receive low pay. Thus, controlling for environmental influences might be helpful better to assess the unique role of values in these relationships. Despite these limitations, we reinforce that our results provide a comprehensive and novel view of the influence of value over work engagement. Such findings can bring new possibilities for future research of values within the workplace.

## Final considerations

Our findings help provide a more detailed understanding of how personal characteristics such as human values influence teachers’ motivation to work. More specifically, all value subfunctions were positively and significantly associated with at least one of the three dimensions of work engagement. However, most importantly, interactive values were the only ones to significantly predict vigor, dedication, and absorption within the workplace. Individuals that endorse this value subfunction are likely to prioritize their interpersonal relationships. Our results show that being motivated by such social values are more likely to help teachers to keep themselves motivated in the workplace in Brazil. Such findings can help develop workplace interventions that highlight the importance of social interactions and promote more significant relationships between teachers and their peers and students, through social support, affect, and a greater sense of belonging. This might be useful, especially in settings such as Brazil, where challenges in infrastructure are common (Sampaio et al., 2002). For instance, social support among students and teachers might facilitate knowledge sharing (Arpaci & Baloğlu, 2016). Finally, we are confident that our research brings novelty to the literature on work engagement, providing the first assessment of human values’ impact on teachers’ motivation towards work.

## Data Availability

Data is available upon request to Dr. Gabriel Coelho.

## Code availability

Figure’s code is available upon request to Dr. Coelho.
